# The NLRP3 inflammasome modulates glycolysis by increasing PFKFB3 in an IL-1β-dependent manner in macrophages

**DOI:** 10.1038/s41598-019-40619-1

**Published:** 2019-03-11

**Authors:** Orla M. Finucane, Jamie Sugrue, Ana Rubio-Araiz, Marie-Victoire Guillot-Sestier, Marina A. Lynch

**Affiliations:** Neuroinflammatory Research Group, Trinity College Institute of Neuroscience, Trinity College, Dublin 2, Ireland

## Abstract

Inflammation and metabolism are intricately linked during inflammatory diseases in which activation of the nucleotide-binding domain–like receptors Family Pyrin Domain Containing 3 (NLRP3) inflammasome, an innate immune sensor, is critical. Several factors can activate the NLRP3 inflammasome, but the nature of the link between NLRP3 inflammasome activation and metabolism remains to be thoroughly explored. This study investigates whether the small molecule inhibitor of the NLRP3 inflammasome, MCC950, modulates the lipopolysaccharide (LPS) -and amyloid-β (Aβ)-induced metabolic phenotype and inflammatory signature in macrophages. LPS + Aβ induced IL-1β secretion, while pre-treatment with MCC950 inhibited this. LPS + Aβ also upregulated IL-1β mRNA and supernatant concentrations of TNFα, IL-6 and IL-10, however these changes were insensitive to MCC950, confirming that MCC950 specifically targets inflammasome activation in BMDMs. LPS + Aβ increased glycolysis and the glycolytic enzyme, PFKFB3, and these effects were decreased by MCC950. These findings suggest that NLRP3 inflammasome activation may play a role in modulating glycolysis. To investigate this further, the effect of IL-1β on glycolysis was assessed. IL-1β stimulated glycolysis and PFKFB3, mimicking the effect of LPS + Aβ and adding to the evidence that inflammasome activation impacts on metabolism. This contention was supported by the finding that the LPS + Aβ-induced changes in glycolysis and PFKFB3 were attenuated in BMDMs from NLRP3-deficient and IL-1R1-deficient mice. Consistent with a key role for PFKFB3 is the finding that the PFKFB3 inhibitor, 3PO, attenuated the LPS + Aβ-induced glycolysis. The data demonstrate that activation of the NLRP3 inflammasome, and the subsequent release of IL-1β, play a key role in modulating glycolysis via PFKFB3. Reinstating metabolic homeostasis by targeting the NLRP3 inflammasome-PFKFB3 axis may provide a novel therapeutic target for treatment of acute and chronic disease.

## Introduction

The production and release of interleukin-1β (IL-1β) rely on activation of the inflammasome, which has been described as the cytosolic regulator of inflammation. Several inflammasomes have been identified but the best characterized is the nucleotide-binding domain–like receptor Family Pyrin Domain Containing 3 (NLRP3) inflammasome that is key to production of IL-1β by macrophages and other cells. It requires two signals to become activated; one that increases expression of IL-1β mRNA and components of the inflammasome, often induced by Toll-like receptor (TLR) activation, and the second to trigger assembly of the inflammasome which results in activation of caspase 1 and processing and release of IL-1β^1^. Here lipopolysaccharide (LPS) was used as signal 1 while signal 2 was amyloid-β (Aβ); Aβ is phagocytosed by macrophages and microglia, resulting in assembly of the inflammasome because it triggers release of cathepsin B^2^. We have shown that LPS + Aβ induces inflammasome activation in microglia^3^.

A link between the inflammatory profile and the metabolic signature of macrophages has become increasingly clear in the past few years. Specifically it has been shown that, when macrophages are triggered to adopt an inflammatory state, their metabolic profile changes, switching from oxidative to glycolytic metabolism^4^. It is believed that this switch is, at least in part, driven by the need to generate ATP rapidly to support the cells in carrying out their immune function. The evidence has suggested that glycolytic, M1 macrophages express increased 6-phosphofructo-2-kinase/fructose-2,6-bisphosphatase 3 (PFKFB3)^5^. This enzyme is a potent driver of glycolysis because its overwhelming kinase activity^6^ leads to production of fructose 2,6-bisphosphate, which activates phosphofructokinase (PFK1)^7^, the enzyme that catalyses a rate-limiting step in glycolysis, the conversion of fructose-6-phosphate to fructose-1, 6-bisphosphate. In contrast, M2 macrophages express PFKFB1 in which the kinase:phosphatase activity ratio is approximately 1:1 and therefore they lack the drive that biases the cells towards glycolysis. We have recently reported that interferon-γ (IFNγ)-activated microglia become glycolytic and this is associated with upregulation of PFKFB3^8^, an association that is also evident in microglia prepared from adult mice.

A number of recent studies have suggested that inflammasome activation is modulated, and perhaps even activated, by enzymes involved in glycolysis^9^. For example, inhibiting hexokinase 1, the enzyme catalysing the first step in the glycolytic pathway, decreases caspase 1 activation and IL-1β production^10^, while inhibition of pyruvate kinase, muscle (PKM2) which catalyses the last step in the glycolytic pathway, also suppresses NLRP3 inflammasome activation in macrophages^11^. In contrast, others have reported that dissociation of hexokinase from its interaction with a voltage-dependent anion channel on the mitochondrial outer membrane and its subsequent translocation to the cytosol, which is indicative of its inhibition, can initiate inflammasome activation in macrophages^12^. It has also been shown that inhibition of the glycolytic enzymes, glyceraldehyde-3-phosphate dehydrogenase and α-enolase can trigger inflammasome activation^13^. These conflicting data indicate that the nature of the interface between cell metabolism and IL-1β production remains to be clarified.

Here we aimed to dissect the interaction between inflammasome activation and the metabolic signature induced by Aβ in LPS-primed macrophages by assessing the modulatory effect of the small molecule inhibitor of the inflammasome, MCC950. Predictably, MCC950 inhibited the LPS + Aβ-induced production of IL-1β. However it also inhibited the glycolytic response and the LPS + Aβ-induced increase in PFKFB3; these changes were markedly reduced in macrophages from NLRP3^−/−^ mice and attenuated by the cell-permeable PFKFB3 inhibitor, 3PO. The data demonstrate that IL-1β increased glycolysis and that the LPS + Aβ -induced glycolytic response was attenuated in macrophages from IL-1R1^−/−^ mice. The findings suggest that inflammasome activation and, in particular a feedback loop by which IL-1β interacts with IL-1R1, elicits a switch towards glycolysis via PFKFB3 in macrophages.

## Materials and Methods

### Animals

C57BL/6, NLRP3^−/−^ and IL-1R1^−/−^ male mice were used in this study. All experiments were performed under license obtained from the Health Products Regulatory Authority of Ireland in accordance with EU regulations and with local ethical approval (Trinity College Dublin). Animals were housed under controlled conditions (20–22 °C, food and water *ad libitum*) and maintained under veterinary supervision.

### Bone marrow derived macrophages (BMDMs)

BMDMs were isolated from the marrow of the femurs and tibias of 8–12 week old mice as described previously^14^. Cells were flushed into a sterile Falcon tube in serum rich media (Dulbecco’s modified Eagle’s medium-DMEM; Invitrogen, UK, supplemented with 10% foetal bovine serum; Gibco, UK and 1%; penicillin–streptomycin Gibco, UK). The cell suspension was centrifuged (400 × g, 5 min) and the resulting pellet was resuspended in serum-rich media supplemented with 20% L929 conditioned media. Cells were seeded in sterile cell culture T75 cm^2^ flasks, non-adherent cells were removed on day 3, media was replaced, and cells were cultured for a further 4 days. On day 7, cells were seeded (0.5 × 10^6^ cells/ml) and cultured overnight. Four different treatment conditions were used. (1) Cells were primed with LPS (100 ng/ml) for 3 h, pre-treated with MCC950 (100 nM) or PBS for 30 min and incubated with amyloid-β (Aβ_1−40_ (4.2 µM) + Aβ_1−42_ (5.8 µM); hereafter referred to as Aβ). In some experiments, the effect of LPS alone and Aβ alone were assessed. (2) Cells were primed with LPS for 3 h and incubated with Aβ as described in 1 above, media was removed and cells were treated with the cell-permeable PFKFB3 inhibitor; 3PO (50 nM; Merck, Ireland) or the vehicle control, DMSO (Sigma-Alrich, UK) for 2 h. (3) Cells were stimulated with mouse recombinant IL-1β (100 ng/ml; R&D Systems, UK) for 24 h. (4) Cells were primed with LPS for 3 h, pre-treated with MCC950 or PBS for 30 min and incubated with monosodium ureate (MSU; 50 nM) for 15 min. The metabolic profile of cells was assessed using SeaHorse technology (see 2.5), cells were harvested for analysis by PCR or western immunoblotting, and supernatant was collected for cytokine analysis.

### ELISA and RT-PCR

Supernatant samples were assessed for concentrations of IL-1β, TNFα, IL-6 and IL-10 using DuoSet ELISA Development Systems (R&D Systems, UK) as per the manufacturer’s instructions. Absorbance was read at 450 nm using a BioTek Synergy HT microplate reader. mRNA expression of IL-1β, TNFα, IL-6, IL-10, PFKFB3 and PFKFB1 was examined using 7300 Real Time PCR System (Applied Biosystems, UK) using the protocol described by Holland and colleagues^8^.

### Western immunoblotting

BMDMs were prepared for western immunoblotting as previously described^3^. Briefly, cells were harvested in lysis buffer (Tris-HCl 10 mM, NaCl 50 mM, 1% Igepal, 10% phosphatase inhibitor cocktail I and II, and protease inhibitor; Sigma, UK) and protein concentrations in the samples were equalised to 15 µg. Samples were added to 4x Laemmli SDS sample buffer (Tris-HCl 100 mM (pH 6.8) containing 4% SDS, 2% bromophenol blue, 20% glycerol; Sigma, UK), boiled (100 °C, 5 min) and applied to 15% SDS gels. Proteins were transferred to nitrocellulose membrane (0.22 µm) and membranes were incubated in 5% non-fat dried milk to block non-specific binding. Membranes were incubated overnight at 4 °C with antibodies raised against PFKFB1 and PFKFB3 (IgG, raised in rabbit, 1:1000 in 2% BSA/TBS-T; Abcam, UK) and β-actin (IgG raised in mouse, 1:5000 in 5% milk/TBS-T; Sigma-Alrich, UK). Membranes were washed and incubated (room temperature, 2 h) with a secondary HRP-linked anti-rabbit antibody (1:3000 in 3% BSA in TBS-T). Immunoreactive bands were detected using WesternBright ECL chemiluminescent substrate (Advansta, US). Images were captured using a Fujifilm LAS-4000 imager and densitometric analysis was carried out using ImageJ (http://rsb.info.nih.gov/). The results are expressed as fold change in treated cells relative to control cells.

### Metabolic analysis

The SeaHorse Extracellular Flux (XF24) analyser (SeaHorse Bioscience, US) was used to carry out bioenergetic analysis of cells. BMDMs (5 × 10^5^ cells/well) were seeded in SeaHorse cell culture microplates and incubated overnight at 37 °C. BMDMs were stimulated as described in section 2.2. The sensor cartridge was hydrated by adding SeaHorse XF calibrant solution (1 ml; SeaHorse BioScience, US) to each well of the utility plate and left overnight in a CO_2_-free incubator at 37 °C. Cells were washed with the specific assay medium for the glycolytic flux test or the mitochondrial stress test according to the manufacturer’s instructions. For the glycolytic flux test, glucose (10 mM), oligomycin (20 µM) and 2-deoxy-D-glucose (2-DG; 500 mM; all Sigma-Aldrich, UK) were prepared in glycolytic flux assay media and loaded into the appropriate ports for sequential delivery. For the mitochondrial stress test, oligomycin (20 µM; Abcam, UK), carbonyl cyanide-4-(trifluorome thoxy)phenylhydrazone (FCCP; 20 µM; Sigma-Aldrich, UK) and antimycin A (40 µM; Sigma-Aldrich, UK) were prepared in mitostress assay media and loaded into the appropriate ports. The extracellular acidification rate (ECAR) and the oxygen consumption rate (OCR) were measured every 7 min and the appropriate compounds were injected sequentially at 24 min intervals. ECAR and OCR were automatically calculated using the SeaHorse XF24 software.

### Statistical analysis

Data are reported as the mean ± SEM and the number of experiments is indicated in each case. Statistical analysis was carried out using a two-way analysis of variance (ANOVA), with *post hoc* Bonferroni tests, or two-tailed student t-test, as appropriate, using Graphpad Prism 5 (GraphPad Software, US). The significance level was set at p < 0.05.

## Results

### MCC950 inhibits the LPS + Aβ-induced NLRP3 inflammasome activation in macrophages

**Figure 1:**
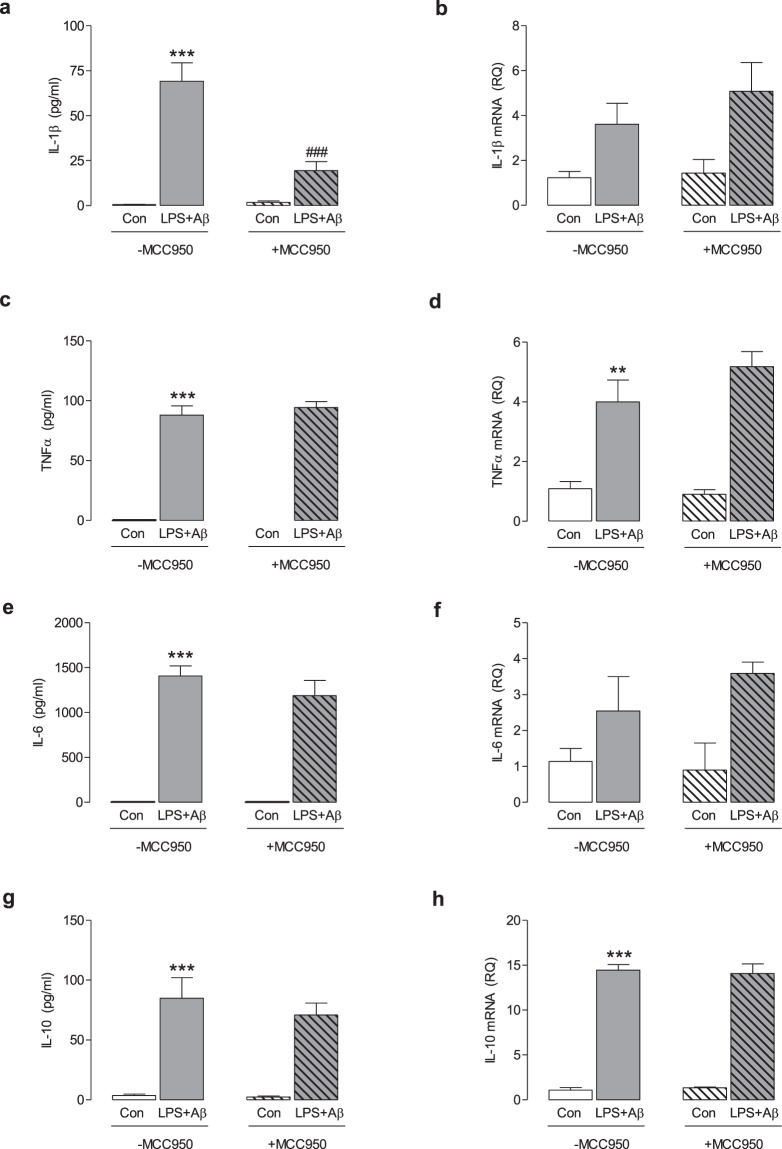
MCC950 impedes NLRP3 inflammasome activation in response to LPS + Aβ. BMDMs, isolated from the femurs and tibias of mice, were cultured for 7 days as described in the Methods (section 2.2). BMDMs were primed with LPS (100 ng/ml) for 3 h, pre-treated with MCC950 (100 nM) for 30 min and subsequently incubated with Aβ (10 µM) for 24 h. Cell supernatants were collected for analysis by ELISA or harvested for RT-PCR analysis. LPS + Aβ increased IL-1β (**a**), IL-1β mRNA (**b**), TNFα (**c**), TNFα mRNA (**d**), IL-6 (**e**), IL-6 mRNA (**f**), IL-10 (**g**) and IL-10 mRNA (**h**) (**p < 0.01, ***p < 0.001; Con vs LPS + Aβ). MCC950 decreased the LPS + Aβ-induced IL-1β secretion (a; ^###^p < 0.001; LPS + Aβ vs LPS + Aβ + MCC950). MCC950 has no effect on any other LPS + Aβ-induced changes. Data are expressed as the mean ± S.E.M. and analysed using a two-way ANOVA and Bonferroni post hoc test; n = 6–7 mice/group.

Previously-published data generated by our laboratory group demonstrated that MCC950 inhibited LPS + Aβ-induced NLRP3 inflammasome activation in microglia^3^. Correspondingly, in this study LPS + Aβ increased supernatant concentrations of IL-1β (***p < 0.001, Fig. 1a) in BMDMs. MCC950 significantly attenuated this LPS + Aβ-induced change (^###^p < 0.001, Fig. 1a). LPS + Aβ increased mRNA expression of IL-1β, TNFα, IL-6 and IL-10 (**p < 0.01; ***p < 0.001) and supernatant concentrations of TNFα, IL-6 and IL-10 (***p < 0.001, Fig. 1b–h). MCC950 did not affect these LPS + Aβ-induced changes, indicating that MCC950 specifically targeted the inflammasome.

### The LPS + Aβ-induced increase in glycolysis is attenuated by MCC950

**Figure 2:**
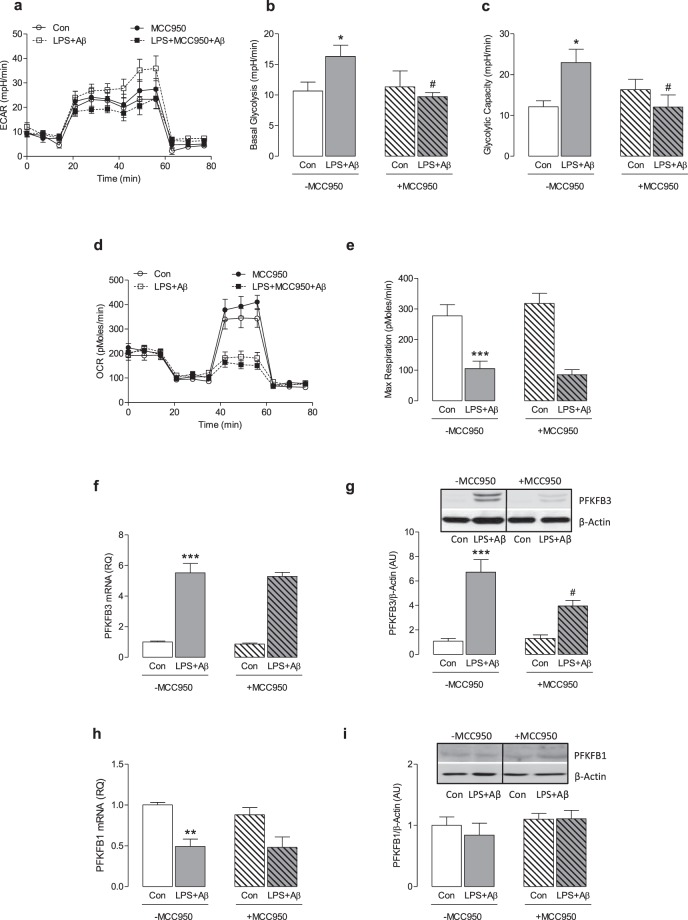
MCC950 impedes glycolysis and reduces PFKFB3 protein expression. BMDMs were stimulated as described in Fig. 1. Metabolic analysis was carried out using the SeaHorse Extracellular Flux (XF24) Analyser and cells were harvested for analysis by western immunoblotting. LPS + Aβ increased extracellular acidification rate (ECAR), basal glycolysis and glycolytic capacity (**a**–**c**; *p < 0.05; Con vs LPS + Aβ, n = 3 mice/group), while MCC950 decreased these effects (**b**,**c**; ^#^p < 0.05; LPS + Aβ vs LPS + Aβ + MCC950). LPS + Aβ decreased oxygen consumption rate (OCR) and reduced maximum respiration (**d**,**e**; ***p < 0.001; Con vs LPS + Aβ, n = 3 mice/group). MCC950 had no effect on OCR or maximum respiration. LPS + Aβ increased PFKFB3 mRNA in BMDMs (**f**; ***p < 0.001; Con vs LPS + Aβ, n = 4 mice/group) but MCC950 had no effect on the LPS + Aβ-induced changes. LPS + Aβ increased PFKFB3 as demonstrated in the representative immunoblot and by analysis of the mean densitometry data (**g**; ***p < 0.001; Con vs LPS + Aβ); MCC950 decreased this effect (^#^p < 0.05; LPS + Aβ vs LPS + Aβ + MCC950, n = 7–8 mice/group) (Original blot Supplementary Fig. 3a). LPS + Aβ decreased PFKFB1 mRNA (**h**; **p < 0.01; Con vs LPS + Aβ, n = 4 mice/group) however MCC950 had no effect on this LPS + Aβ-induced change. No change in PFKFB1 was observed in LPS + Aβ- or LPS + Aβ + MCC950-treated cells (**i**) (Original blot Supplementary Fig. 3b). Data are expressed as the mean ± S.E.M. and analysed using a two-way ANOVA and Bonferroni post hoc test.

We next examined the impact of LPS + Aβ on cell metabolism. LPS + Aβ increased ECAR as indicated by the metabolic profile obtained from analysis using the SeaHorse glycolytic stress test (Fig. 2a). Analysis of the mean data indicated that basal glycolysis and glycolytic capacity were significantly increased by LPS + Aβ (*p < 0.05; Fig. 2b,c), while MCC950 significantly attenuated these LPS + Aβ-induced changes (^#^p < 0.05). LPS alone and Aβ alone increased ECAR and basal glycolysis, while MCC950 did not modulate either the LPS- or Aβ-induced changes (Supplementary Fig. 1a–c). To investigate whether MCC950 modulates glycolysis induced by other stimuli that activate the inflammasome, we assessed its impact on MSU-induced ECAR in LPS-primed macrophages. The data show that MSU increased ECAR and basal glycolysis but MCC950 had no effect on these changes (Supplementary Fig. 2a,b). LPS + Aβ decreased OCR as demonstrated by the metabolic profile of cells (Fig. 2d) and significantly reduced maximum respiration (***p < 0.001; Fig. 2e) but MCC950 exerted no modulatory effect on these measures. To explore the mechanism by which inhibiting the inflammasome might impact on the metabolic profile of cells, we investigated the effect of LPS + Aβ in the presence or absence of MCC950 on PFKFB3, an enzyme that has been identified as a major driver of glycolysis. LPS + Aβ significantly increased PFKFB3 mRNA (***p < 0.001; Fig. 2f) and also significantly increased PFKFB3 protein as indicated in the sample immunoblot (Fig. 2g, full blot depicted in Supplementary Fig. 3a) and by the mean data obtained from densitometric analysis (***p < 0.001; Fig. 2h). MCC950 exerted no effect on the LPS + Aβ-induced increase in PFKFB3 mRNA (Fig. 2f) but significantly attenuated the increase in PFKFB3 protein (^#^p < 0.05; Fig. 2g). LPS + Aβ significantly decreased PFKFB1 mRNA (**p < 0.01; Fig. 2h), but it did not affect PFKFB1 protein as indicated by the mean data obtained from immunoblotting (Fig. 2i, full blot depicted in Supplementary Fig. 3b). No modulatory effect of MCC950 on PFKFB1 was observed. These findings suggest that inhibiting the inflammasome modulates the glycolytic profile of cells because of its impact on PFKFB3.

### Lack of NLRP3 modulates glycolysis in macrophages

**Figure 3:**
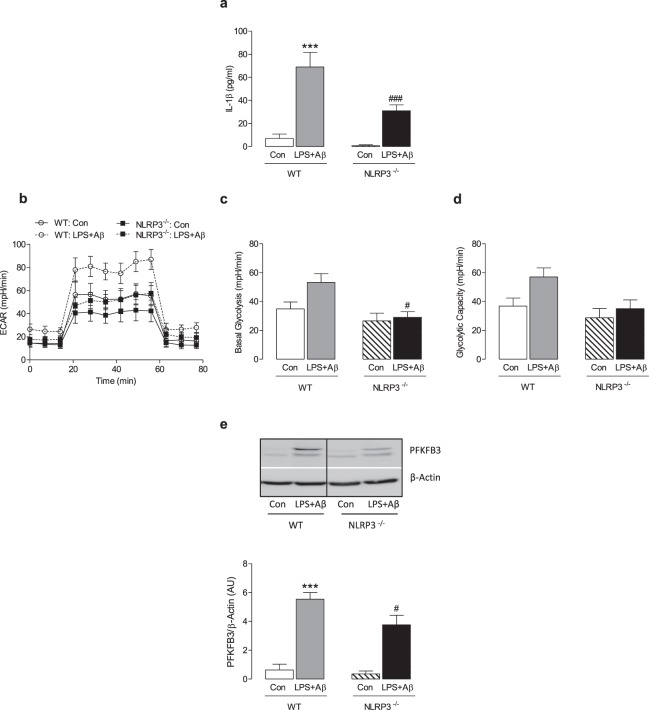
NLRP3 deficiency attenuates the effect of LPS + Aβ on glycolysis and PFKFB3. BMDMs from WT and NLRP3^−/−^ mice were primed with LPS (100 ng/ml) for 3 h, and incubated with Aβ (10 µM) for 24 h. Cell supernatants were collected for analysis by ELISA. LPS + Aβ induced IL-1β production in BMDMs from WT mice (**a**; ***p < 0.001; WT Con vs WT LPS + Aβ, n = 8 mice/group). IL-1β production, in response to LPS + Aβ, was decreased in BMDMs from NLRP3^−/−^, compared with WT, mice (**a**; ^###^p < 0.001; WT LPS + Aβ vs NLRP3^−/−^ LPS + Aβ, n = 8 mice/group). Metabolic analysis was carried out using the SeaHorse Extracellular Flux (XF24) Analyser. The effect of LPS + Aβ on ECAR and basal glycolysis was decreased in BMDMs from NLRP3^−/−^ mice (**b**,**c**; ^#^p < 0.05; WT LPS + Aβ vs NLRP3^−/−^ LPS + Aβ, n = 8–16 mice/group). No change in glycolytic capacity was observed (**d**). LPS + Aβ increased PFKFB3 in BMDMs from WT mice (**e**; ***p < 0.001; Con vs LPS + Aβ, n = 4 mice/group). PFKFB3 was decreased in NLRP3^−/−^ BMDMs compared with WT as demonstrated by the representative blot and the mean densitometric data (**e**; ^#^p < 0.05; WT LPS + Aβ vs NLRP3^−/−^ LPS + Aβ) (Original blot presentation Supplementary Fig. 4). Data are expressed as the mean ± S.E.M. and analysed using a two-way ANOVA and Bonferroni post hoc test.

Next we compared the effect of LPS + Aβ on IL-1β release, glycolysis and PFKFB3 in BMDMs from WT and NLRP3^−/−^ mice. Consistent with the data in Fig. 1, LPS + Aβ significantly increased IL-1β release (***p < 0.001; Fig. 3a) and this effect was attenuated in BMDMs from NLRP3^−/−^ mice (^###^p < 0.001; Fig. 3a). LPS + Aβ increased the rate of glycolysis, basal glycolysis and glycolytic capacity as previously observed and the response to LPS + Aβ was reduced in BMDMs from NLRP3^−/−^ mice compared with WT mice (^#^p < 0.05; Fig. 3b–d). Immunoblot analysis demonstrated that the significant LPS + Aβ-induced increase in PFKFB3 in WT mice (***p < 0.001; Fig. 3e, full blot depicted in Supplementary Fig. 4) was reduced in BMDMs from NLRP3^−/−^ mice (^#^p < 0.05).

### The NLRP3 inflammasome modulates glycolysis via the IL-1β-PFKFB3 axis

**Figure 4:**
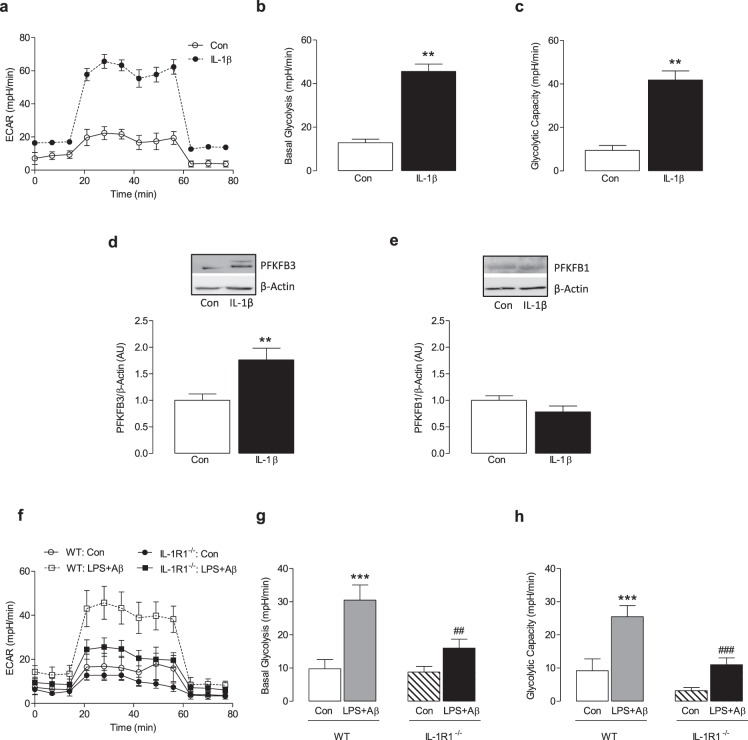
IL-1β increases glycolysis and PFKFB3. BMDMs were stimulated with IL-1β (100 ng/ml) for 24 h. Metabolic analysis was carried out using the SeaHorse Extracellular Flux (XF24) Analyser. IL-1β increased ECAR, basal glycolysis and glycolytic capacity (**a**–**c**; **p < 0.01; Con vs IL-1β, n = 3 mice/group). IL-1β increased PFKFB3 as demonstrated by the representative blot and the mean densitometric data (**d**; **p < 0.01; Con vs IL-1β, n = 3 mice/group) (Original blot Supplementary Fig. 5a,c). IL-β had no effect on PFKFB1 (**e**) (Original blot Supplementary Fig. 5b,c). BMDMs from WT and IL-1R1^−/−^ mice were primed with LPS (100 ng/ml) for 3 h, and incubated with Aβ (10 µM) for 24 h. In response to LPS + Aβ, ECAR, basal glycolysis and glycolytic capacity were increased in cells from WT mice (**f**–**h**; ***p < 0.01; Con vs LPS + Aβ; n = 7–8 mice/group). ECAR, basal glycolysis and glycolytic capacity were significantly decreased in BMDMs from IL-1R1^−/−^ mice compared with WT BMDMs (**f**–**h**; ^##^p < 0.01, ^###^p < 0.001; WT LPS + Aβ vs IL-1R1^−/−^ LPS + Aβ, n = 7–8 mice/group). Data are expressed as the mean ± S.E.M. and assessed using a two-tailed student t-test or, where appropriate, a two-way ANOVA and Bonferroni post hoc test.

**Figure 5:**
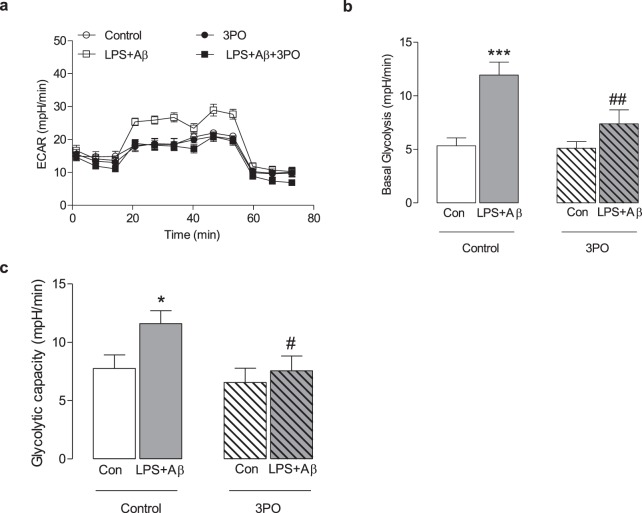
Inhibiting PFKFB3 decreases LPS + Aβ-induced glycolysis. BMDMs were stimulated with LPS + Aβ for 24 h followed by incubation with 3PO for 2 h. Metabolic analysis was carried out using the SeaHorse Extracellular Flux (XF24) Analyser. LPS + Aβ increased ECAR, basal glycolysis and glycolytic capacity (*p < 0.05; ***p < 0.001; **a**–**c**) and 3PO reduced these LPS + Aβ-induced changes (^#^p < 0.01; ^##^p < 0.01). Data are expressed as the mean ± S.E.M. and assessed using a two-way ANOVA and Bonferroni post hoc test.

One possible mechanism by which the inflammasome exerts its effect on metabolism might result from the released IL-1β feeding back to interact with IL-1R1 in an autocrine manner. To investigate this possibility, macrophages were stimulated with IL-1β for 24 h and the metabolic profile of the cells was assessed. IL-1β increased ECAR (Fig. 4a) and, correspondingly, significant increases in mean basal glycolysis and glycolytic capacity were observed (**p < 0.01; Fig. 4b,c). IL-1β also significantly increased PFKFB3 as indicated by the sample immunoblot (Fig. 4d, full blot depicted in Supplementary Fig. 5a) and the mean data obtained from densitometry analysis (**p < 0.01; Fig. 4d). IL-1β had no effect on PFKFB1 (Fig. 4e, full blot depicted in Supplementary Fig. 5b). To investigate whether IL-1β exerts its effect on metabolism by acting through IL-1R1, we examined the glycolytic profile in BMDMs from WT and IL-1R1^−/−^ mice in response to LPS + Aβ. The data show that LPS + Aβ increased ECAR, basal glycolysis and glycolytic capacity in BMDMs from WT mice (***p < 0.001; Fig. 4e–h). The rate of glycolysis in response to LPS + Aβ was reduced in BMDMs from IL-1R1^−/−^ mice compared with WT mice as demonstrated by reduced ECAR, basal glycolysis and glycolytic capacity (^##^p < 0.01; ^###^p < 0.001; Fig. 4e–h). To investigate the importance of PFKFB3 in modulating NLRP3 inflammasome-induced glycolysis BMDMs were treated with the PFKFB3 inhibitor, 3PO. As before, LPS + Aβ increased ECAR, basal glycolysis and glycolytic capacity (*p < 0.05; ***p < 0.001; Fig. 5a–c) and 3PO reduced these LPS + Aβ-induced changes (^#^p < 0.01; ^##^p < 0.01).

## Discussion

Activation of the NLRP3 inflammasome is potentially the central event that links immune function, inflammation and metabolism. Here we demonstrated that the small molecule inhibitor of the NLRP3 inflammasome, MCC950, inhibited LPS + Aβ-triggered glycolysis and the expression of the glycolytic regulator, PFKFB3. This effect was mediated, at least in part, by IL-1β as IL-1β increased glycolysis and PFKFB3, while lack of IL-1 signalling decreased LPS + Aβ induced glycolysis. Our study identifies the NLRP3 inflammasome-IL-1β-PFKFB3 axis as a key regulator of glycolysis in macrophages.

We utilised MCC950 as a tool to manipulate the NLRP3 inflammasome and thereby investigate the relationship between NLRP3 inflammasome activation and cell metabolism. MCC950 is a potent and selective small molecule that inhibits both the canonical and the non-canonical activation of the NLRP3 inflammasome^15^. It has been shown to attenuate the progressive deterioration in motor function in experimental autoimmune encephalomyelitis, a mouse model of multiple sclerosis^15^ and the amyloid pathology and deterioration in cognition in a transgenic mouse model of Alzheimer’s disease^3^. Here we found that MCC950 decreased Aβ-induced IL-1β production in LPS-primed macrophages mirroring its effect in microglia^3^. However MCC950 had no effect on the LPS + Aβ-induced production of IL-6, TNFα, IL-10 or IL-1β gene expression in macrophages; this is consistent with previous findings^15^ and confirms that MCC950 specifically inhibits the NLRP3 inflammasome.

Inflammatory stimuli trigger metabolic reprogramming in macrophages such that the cells adopt a glycolytic phenotype. It is suggested that the increased rate of glycolysis triggered, for example by PAMPs in response to an infection, rapidly generates ATP to support macrophage immune function^4^. Consistent with this, we show that LPS + Aβ increased the rate of glycolysis and decreased oxidative phosphorylation in BMDMs. It has been suggested that a shift to glycolysis may contribute to inflammasome activation as a result of increased activity of the glycolytic enzymes, hexokinase 1 and PKM2. Specifically, hexokinase 1 activity, under the regulation of mTORC1, induces NLRP3 inflammasome activation^10^ and PKM2 impacts on NLRP3 and AIM2 inflammasome activation as a result of modulating phosphorylation of eukaryotic translational initiation factor 2 alpha kinase 2 (EIF2AK2)^11^. Indeed a crucial role for EIF2AK2 in inflammasome activation has been described and genetic deletion of EIF2AK2 markedly reduces IL-1β release^16^.

Significantly, we show that MCC950 decreased glycolysis in LPS + Aβ-treated macrophages without affecting oxidative phosphorylation. This suggests that activation of the NLRP3 inflammasome can modulate glycolysis and perhaps can reinstate metabolic equilibrium. To address this, we repeated the assessments in macrophages harvested from NLRP3^−/−^ mice. Both IL-1β production and basal glycolysis were decreased in LPS + Aβ-stimulated macrophages from NLRP3^−/−^ mice compared with macrophages from WT mice, although the ablation of IL-1β production or glycolysis was incomplete. These data, and the data demonstrating the stimulatory effect of glycolytic enzymes on NLRP3 activation, suggest that a modulatory loop between inflammasome activation and metabolic status determines the reprogramming of cells.

PFKFB3 is a key enzyme in the regulation of glycolysis. It fuels the proliferation of cancer cells by regulating glycolysis^17^ and its activity in macrophages is critical in mounting immune defence^18^, whereas inhibiting PFKFB3 prohibited engulfment and removal of virus-infected cells^19^. Here we show that LPS + Aβ increases PFKFB3 expression and a similar increase also accompanied the IFNγ-stimulated glycolysis in microglia^8^. An important finding in the present study is that MCC950 decreased the LPS + Aβ-induced increase in PFKFB3, while the effect of LPS + Aβ on PFKFB3 in macrophages from NLRP3^−/−^ mice was attenuated. A positive correlation between PFKFB3 and IL-1β production was observed. Thus we hypothesised that NLRP3 inflammasome may mediate its effects on glycolysis by means of an IL-1β-PFKFB3 axis and therefore PFKFB3 may be a plausible target for modulating the NLRP3 inflammasome. Information regarding the direct impact of IL-1β on cell metabolism is sparse, particularly in macrophages, although it has been suggested that it may act as a metabolic hormone in the ovary because it stimulates glycolysis in rat ovarian cells apparently producing lactate from the available glucose^20^.

To determine whether IL-1β might be a factor in driving glycolysis and might potentially explain the key role of the NLRP3 inflammasome in modulating the metabolic signature of macrophages, we stimulated cells with recombinant IL-1β. IL-1β mimicked the effect of LPS + Aβ and increased glycolysis and PFKFB3. Moreover the impact of LPS + Aβ on glycolysis was markedly reduced in macrophages derived from IL-1R1^−/−^ mice. Importantly, inhibiting PFKFB3 activity impeded the LPS + Aβ-induced increase in glycolysis. These data identify IL-1β as an important modulatory cytokine in metabolic control and suggest that it is the key to the link between the NLRP3 inflammasome and glycolysis.

### Conclusion

Metabolic reprogramming of macrophages is necessary for macrophages to adequately respond to invading pathogens. However during acute and chronic inflammatory diseases the cells ability to reinstate metabolic homeostasis is lost. We demonstrate that the NLRP3 inflammasome is an important regulator of glycolysis. Our evidence suggests that the NLRP3 inflammasome mediates its modulatory actions via IL-1β-PFKFB3 axis and targeting this axis may facilitate resolution of inflammation and restore metabolic balance in macrophages.

## Supplementary information

Supplementary Figure

## Data Availability

The datasets used or analysed during the current study are available from the corresponding author on reasonable request.
