# Supplementary material for: The NLRP3 inflammasome modulates glycolysis by increasing PFKFB3 in an IL-1β-dependent manner in macrophages
Source: Sci Rep. 2019 Mar 11;9:4034. doi: 10.1038/s41598-019-40619-1 (PMC6411754; doi:10.1038/s41598-019-40619-1)
Supplement: Supplementary file 1 — Supplementary Figure [file 41598_2019_40619_MOESM1_ESM.pdf]

The NLRP3 inflammasome modulates glycolysis by increasing PFKFB3 in an IL-1β-dependent manner in macrophages

Orla M. Finucane*, Jamie Sugrue, Ana Rubio-Araiz, Marie-Victoire Guillot-Sestier, Marina A. Lynch
Neuroinflammatory Research Group,
Trinity College Institute of Neuroscience,
Trinity College,
Dublin 2

*Corresponding author
Email: finucaor@tcd.ie
Address: Trinity College Institute of Neuroscience,
Lloyd Building, Trinity College Dublin,
Dublin 2, Ireland.
Tel: 353-1-896-8476.
Fax: 353-1-896-3545.

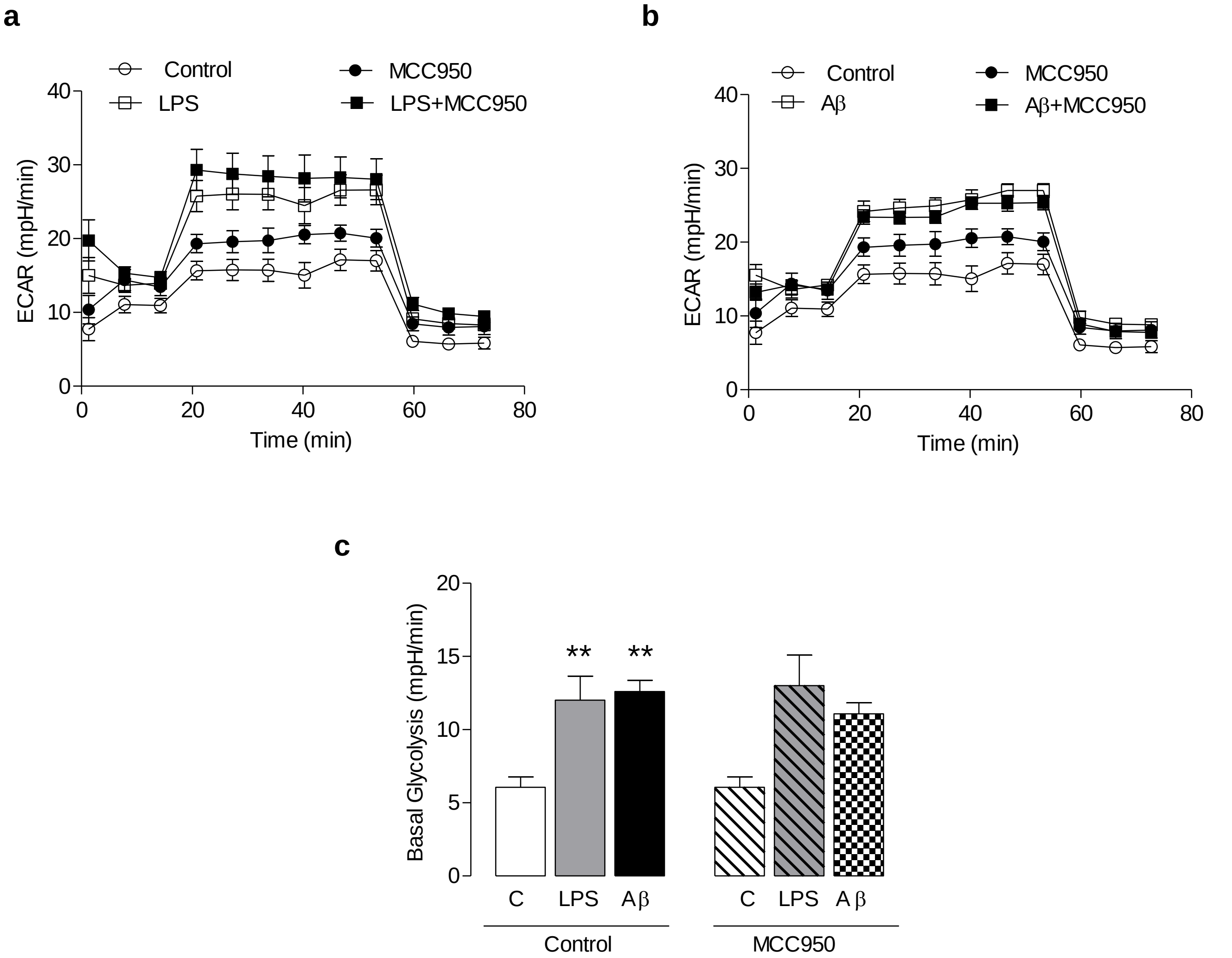

Supplementary Fig 1. MCC950 does not attenuates LPS or Aβ induced metabolic changes
BMDMs, isolated from the femurs and tibias of mice, were cultured for 7 days as described in the Methods (section 2.2). BMDMs were stimulated with LPS (100 ng/ml) for 3 h or Aβ (10 µM) for 24 h. Metabolic analysis was carried out using the SeaHorse Extracellular Flux (XF24) Analyser as described in Methods (section 2.5). LPS alone and Aβ alone increased extracellular acidification rate (ECAR) and basal glycolysis (a-c; $^{**}p < 0.01$; Con vs LPS, Con vs Aβ n=3 mice/group). MCC950 had no effect on these changes.

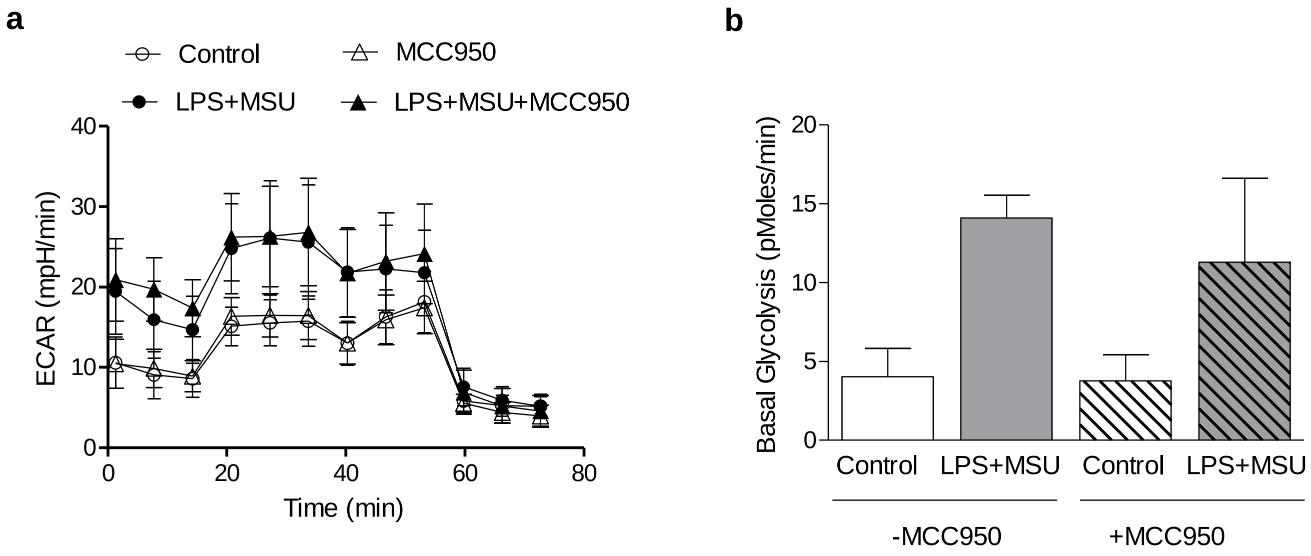

Supplementary Fig 2. MCC950 does not attenuate LPS+MSU induced metabolic changes
BMDMs were stimulated with LPS (100 ng/ml) for 3 h or MSU (50 nM) for 15 min. Metabolic analysis was carried out using the SeaHorse Extracellular Flux (XF24) Analyser as described in Methods (section 2.5). LPS+MSU increased extracellular acidification rate (ECAR) and basal glycolysis (a-b; **$p < 0.01$; Con vs LPS+MSU). MCC950 had no effect on these changes.

a

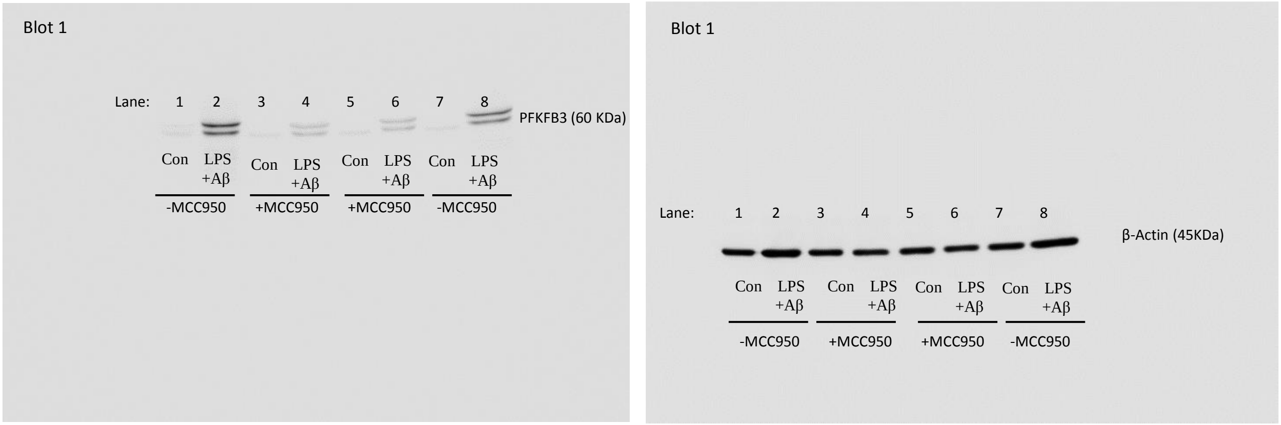

b

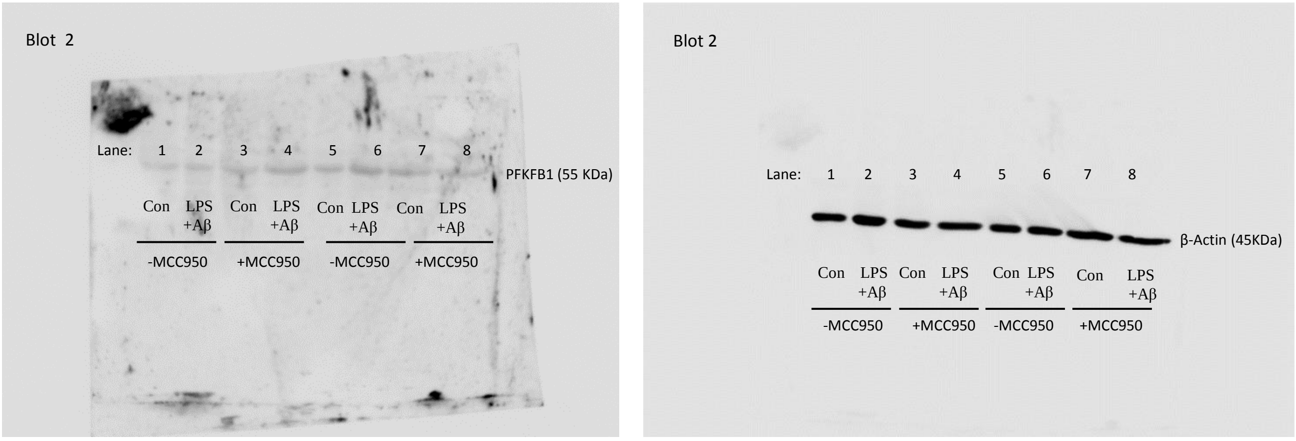

Supplementary Fig 3. MCC950 reduces PFKFB3 protein expression.
Cells were lysed and a total of 15µg of protein was loaded onto each gel. The same samples were loaded onto blot 1 and blot 2. The images were captured using the Fujifilm LAS-4000 imager, cropped using Microsoft Office Picture Manager 2010, changed to grey background using Microsoft PowerPoint. (a) Original Blot 1: depicting protein levels of PFKFB3 and β-Actin, lanes 1-4 were chosen as the best representation of results and inserted into Fig. 2g of the main article. (b) Original Blot 2: depicting protein levels of PFKFB1 and β-Actin, lanes 1-4 were chosen as the best representation of results and inserted into Fig. 2i of the main article.

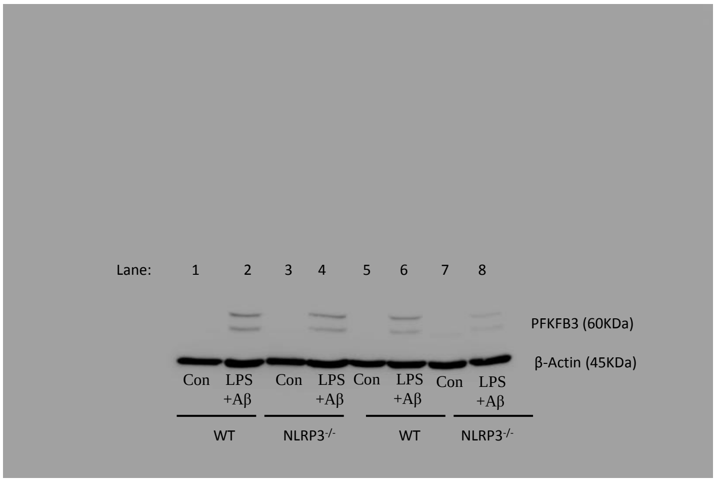

Supplementary Fig 4. NLRP3 deficiency attenuates the effect of LPS+Aβ on PFKFB3. BMDMs, isolated from the femurs and tibias of mice, were cultured for 7 days as described in the Methods (section 2.2). BMDMs were primed with LPS (100 ng/ml) for 3 h, pre-treated with MCC950 (100 nM) for 30 min and subsequently incubated with Aβ (10 µM) for 24 h. Cells were lysed and a total of 15 µg of protein was loaded onto each gel. The image were captured using the Fujifilm LAS-4000 imager, cropped using Microsoft Office Picture Manager 2010, changed to appropriate grey background using Microsoft PowerPoint. (a) Original blot depicting protein levels of PFKFB3 and loading control β-Actin, lanes 5-8 were chosen as the best representation of results and inserted into Fig. 3e of the main article.

a

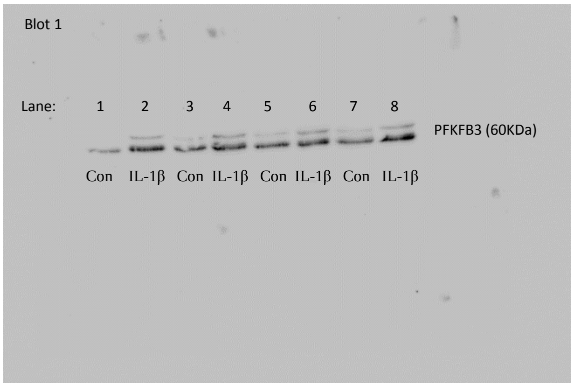

b

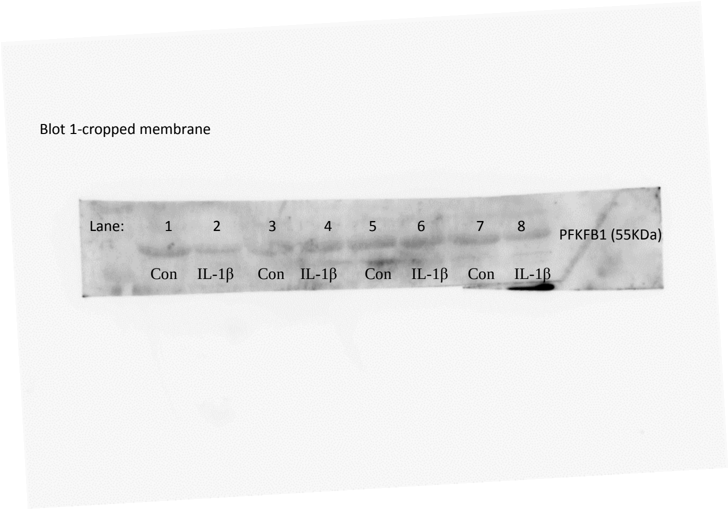

c

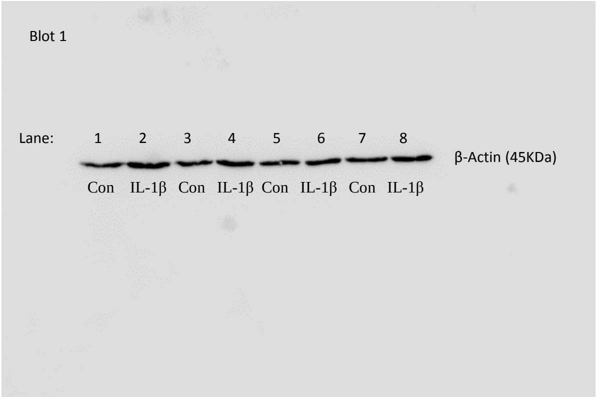

Supplementary Fig 5. IL-1β increases PFKFB3. BMDMs were stimulated with recombinant IL-1β (100 ng/ml) for 24 h. Cells were lysed and a total of 15 µg of protein was loaded onto a 15% gel. The images were captured using the Fujifilm LAS-4000 imager, cropped using Microsoft Office Picture Manager 2010, changed to appropriate grey background using Microsoft PowerPoint. PFKFB3, PFKFB1 and β-Actin were imaged on the same blot on separate days. (a) Depicts protein levels of PFKFB3, lanes 1 and 2 were chosen as the best representation of the results and inserted into Figure 4d of the main article. (b) The original blot was cut and PFKFB1 imaged, lanes 7 and 8 were chosen as the best representation of results and inserted into Fig. 4e of the main article. (c) The corresponding loading control for Blot 1, β-Actin. Lanes 1-2 were cropped and inserted into Fig. 4d of the main article while lanes 7 and 8 were cropped and inserted into Fig. 4e of the main article.
